# *Plectranthus amboinicus* and rosemary (*Rosmarinus officinalis* L.) essential oils effects on performance, antioxidant activity, intestinal health, immune response, and plasma biochemistry in broiler chickens

**DOI:** 10.1002/fsn3.3380

**Published:** 2023-04-21

**Authors:** Saied Hosseinzadeh, Farid Shariatmadari, Mohammad Amir Karimi Torshizi, Hamed Ahmadi, Dawn Scholey

**Affiliations:** ^1^ Department of Poultry Science, Faculty of Agriculture Tarbiat Modares University Tehran Iran; ^2^ School of Animal Rural & Environmental Sciences Nottingham Trent University Nottingham UK

**Keywords:** antioxidant, broiler chicken, intestinal microbiota, intestinal morphology, performance, *Plectranthus amboinicus*

## Abstract

This work aimed to assess the effects of *Plectranthus amboinicus* essential oil (PAE) and rosemary (*Rosmarinus officinalis* L.) essential oil (ROE) as feed additives on performance, antioxidant activity, intestinal microbiota, intestinal morphology, immune response, and plasma biochemistry using 320 unsexed 1‐day‐old Ross 308 broiler chickens. The chickens were assigned randomly into four treatments containing eight replicates with 10 chickens each. Treatment diets included a basal diet as a control group, 100 mg/kg PAE, 200 mg/kg PAE, and 100 mg/kg ROE. ROE affected the growth performance in the starter phase by improving (*p* = .01) the feed conversion ratio (FCR) compared with the control diet. Glutathione peroxidase (GPx) and superoxide dismutase (SOD) activity in the plasma were elevated (*p* < .0001) by both feed additives. Supplementation of additives could increase (*p* < .006) total antioxidant capacity (TAC). Furthermore, malondialdehyde (MDA) values in the breast (*p* < .0001) and thigh (*p* < .001) for all supplemented diets were less than the control group. The essential oils (EOs) reduced (*p* < .005) coliform counts in the ileum and increased (*p* = .029) lactic acid bacteria counts. In addition, villus height (VH) and crypt depth (CD) increased, whereas the density of goblet cells decreased in the small intestine when feed additives were included. Also, the antibody titers against sheep red blood cells (SRBC) and Newcastle disease virus (NDV) were increased (*p* < .0001) by EOs. Plasma total protein (*p* = .04) and globulin (*p* = .02) were increased, and cholesterol was reduced (*p* = .002) by supplemented diets. Our study revealed that PAE could effectively improve the antioxidant activity, intestinal microbiota population, intestinal morphology, immune response, and plasma biochemistry parameters in broiler chickens.

## Graphical abstract

Our study showed *Plectranthus amboinicus* and Rosemary EOs increased the activity of plasma antioxidant enzymes, decreased MDA in meat, improved intestinal microbiota and morphology, improved humoral immunity, as well as, reduced plasma cholesterol, and increased plasma protein in broiler chicken.

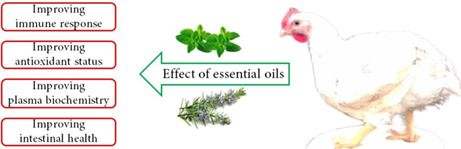

## 1 INTRODUCTION

Phytobiotics, also known as phytogenics or phytochemicals, are an extensive subset of plant‐based bioactive compounds. Over 5000 individual dietary phytobiotics have been recognized to date in fruits, whole grains, vegetables, legumes, herbs, nuts, and EOs (Landy et al., 2011; Liu, 2004). Herbs and EOs from the Lamiaceae family have revealed antioxidant, antibacterial, and immunostimulatory effects and increased enzymatic secretions in the gastrointestinal tract of animals (Brenes & Roura, 2010; Ghalamkari et al., 2011). These can potentially be an alternative to antibiotics, or used alongside (Aguiar et al., 2015). The characteristics of *Plectranthus amboinicus* (Lour.) Spreng (PA), a member of the Lamiaceae family, has been studied for effects including antibacterial (Aguiar et al., 2015), antifungal (Murthy et al., 2009), antivirus (Kusumoto et al., 1995), and antioxidant (Praveena & Pradeep, 2012) activities in vitro. However, there are few studies in vivo, particularly using farm animals. The leaf meal of PA enhanced the milk production of dairy Holstein cows (Fati et al., 2014) and PA extract was shown to have antioxidant, nephroprotective, and diuretic properties (Palani et al., 2010) in rats. Similarly, PAE increased antioxidant activity (Manjamalai & Grace, 2012) in mice. In other species, PA extract improved the nonspecific immune response and growth performance in white‐leg shrimp (Huang et al., 2022). This herbal essential oil has the main bioactive compounds of carvacrol (Murthy et al., 2009) and thymol (Sabra et al., 2018), which can be compared with thyme and oregano EOs. Rosemary (*Rosmarinus officinalis* L.) is another aromatic plant from the Lamiaceae family with the main components including camphor, 1,8‐cineole, and α‐pinene (Sienkiewicz et al., 2013). ROE is more well known than PAE for farm animal research, with numerous studies utilizing ROE as a feed additive in poultry (Gharejanloo et al., 2017; Loetscher et al., 2013; Mahgoub et al., 2019; Mathlouthi et al., 2012; Yesilbag et al., 2011). Therefore, in the current study with PAE, its essential oil potential was assessed at 100 mg/kg, which for ROE was an effective dosage in some studies (Gharejanloo et al., 2017; Mathlouthi et al., 2012; Yesilbag et al., 2011). The present work aimed to determine the effect of feeding PAE compared with rosemary on performance, antioxidant activity, intestinal microbiota, intestinal morphology, immune response, and plasma biochemistry in modern commercial broilers.

## 2 MATERIALS AND METHODS

### 2.1 Preparation and extract of essential oils

The PA was raised in the greenhouse of Tarbiat Modares Agriculture Faculty. We purchased ROE from a local company. The fresh aerial parts of PA were collected and exposed to hydro‐distillation for 3 h via a Clevenger‐type apparatus to extract the essential oil. Dehydrating the essential oil used sodium sulfate, and it was stored after filtration at 4°C and in darkness to safeguard its components.

### 2.2 GC/MS analysis

*Plectranthus amboinicus* essential oil (PAE) and rosemary (*Rosmarinus officinalis* L.) essential oil (ROE) analysis was performed using a Thermoquest‐Finnigan Trace GC–MS tool equipped with a DB‐5 fused silica capillary column (film thickness 0.25 mm, 60 m × 0.25 mm i.d.). The temperature of the oven was programmed to increase from 60 to 250°C at a rate of 4°C per minute. It was ultimately held for 10 min; at a transfer line temperature of 250°C. The carrier gas was helium with a split ratio of 1/50 at a 1.1 mL/min flow rate. Over 35–465 amu (atomic mass units) the mass spectrometer was scanned via the quadrupole, with an ionizing voltage of 70 eV and an ionization current of 150 mA. The constituents were identified based on their retention indices by comparison of the mass spectra with those reported in the studies and Willey (Chem Station data system) libraries (Table 1).

**TABLE 1:** Identification of essential oil components by GC–MS.

*Plectranthus amboinicus* essential oil		Rosemary essential oil	
Compound	(%)	Compound	(%)
α‐Thujene	0.8	α‐Thujene	0.2
α‐Pinene	0.5	α‐Pinene	23.6
1‐Octen‐3‐ol	0.8	Camphene	5.7
δ‐3‐Carene	0.9	β‐Pinene	3.2
α‐Terpinene	2	3‐Octanone	1.8
p‐cymene	7.7	β‐Myrcene	3.5
γ‐Terpinene	18.8	δ‐3‐Carene	0.3
α‐Terpinolene	0.2	Limonene	3.4
Linalool	0.2	1,8‐cineole	12.5
Terpinen‐4‐ol	0.6	α‐Terpinolene	0.6
Thymol	51.6	Linalool	2.3
Carvacrol	1.4	Camphor	9.5
β‐Caryophyllene	6.5	Borneol	6.1
α‐Humulene	0.5	Terpinen‐4‐ol	0.6
α‐Selinene	0.3	Verbenone	4.6
β‐Selinene	2	Bornyl acetate	8.7
Spatulenol	0.4	β‐Caryophyllene	4.5
10‐epi‐γ‐Eudesmol	0.3	Caryophyllene oxide	1.3
τ‐Cadinol	0.6		
α‐Eudesmol	0.4		
Valeranone	0.3		

### 2.3 Animals, diets, and experimental design

This study was conducted at the poultry farm of Tarbiat Modares Agriculture Faculty (latitude 35°44′N; longitude 51°9′E and altitude 1284 m above sea level) using 320 unsexed 1‐day‐old broiler chickens (Ross308, Aviagen Inc.). The chickens were randomly allocated into four treatments with eight replicates of 10 chickens each. The treatments were the basal diet as the control group, basal diet +100 mg/kg PAE, basal diet +200 mg/kg PAE, and basal diet +100 mg/kg ROE. Table 2 shows the nutrient composition and the ingredients of the basal diet. The formulation of diets met the nutrient requirements according to Ross308 rearing guidelines. The chickens were kept in pens (1 m by 1 m) in a ventilated broiler house with wood shavings as litter material. The animals had access to food and water ad libitum during the feeding period of 42 days. The initial temperature of the house was 32°C, which decreased gradually to obtain 20°C by day 27. The lighting program was 23 h light: 1 h dark for the first 7 days and then changed to 19 h light: 5 h dark until the end of the study period. The study protocol was evaluated and accepted by the Tarbiat Modares University's Ethics Committee for animal use.

**TABLE 2:** Ingredients and calculated chemical and energy content of the basal diet.

Item	Starter (days 0–10)	Grower (days 11–24)	Finisher (days 25–42)
Ingredients (%)			
Corn, yellow grain	56	59	62.5
Soybean meal, solvent	37	32	27
Soybean oil	0.85	2.2	3.17
Meat and bone meal	3	5	5
Di‐calcium phosphate	0.95	0.3	0.09
Limestone	0.9	0.62	0.54
Feed salt	0.22	0.2	0.2
Sodium bicarbonate	0.05	0.1	0.3
Premix^a^	0.5	0.5	0.5
L‐lysine HCl	0.07	0.05	0.05
DL‐methionine	0.22	0.2	0.19
Calculated composition			
Metabolizable energy, MJ/kg	12.05	12.72	13.09
Crude protein, %	22.17	21.17	19.22
Calcium, %	0.92	0.85	0.76
Available phosphorus, %	0.46	0.42	0.38
Sodium, %	0.15	0.17	0.18
Dig. Lysine, %	1.22	1.12	0.99
Dig. Methionine, %	0.57	0.53	0.49
Dig. Methionine+ Cystine, %	0.91	0.85	0.78
Dig. Threonine, %	0.80	0.75	0.68

^a^ The vitamin/mineral premix provided the following per kilogram of diet: vitamin A)trans‐retinyl acetate(, 10,000; vitamin D3 (cholecalciferol), 4400 IU; vitamin E (DL‐α‐tocopherol acetate), 27 IU; vitamin K (Menadione), 2.8 mg; thiamine, 1.75 mg; riboflavin, 6.6 mg; pyridoxine, 3 mg; vitamin B12 (cyanocobalamin), 0.015 mg; niacin, 26 mg; calcium pantothenate, 11 mg; folic acid, 1 mg; biotin, 0.1 mg; choline chloride, 250 mg; Fe, 30 mg; Mn, 105 mg; Zn, 73 mg; Cu, 10 mg; I, 1 mg; Se, 0.2 mg.

### 2.4 Growth performance assay

The weight gain and feed intake were assayed per pen at the end of each week for the broiler chickens. Then, the average daily feed intake (ADFI), average daily gain (ADG), and the ratio of those as FCR were calculated for the different phases of starter, grower, and finisher. Chick mortality was reported and considered in calculations for all treatments.

### 2.5 Plasma antioxidant assay

At day 42, the blood of two chickens per pen (16 chickens per treatment) was drawn using heparinized syringes via the jugular vein. The blood plasma was separated to assay the activity of antioxidant enzymes using SOD and GPx commercial kits (Navand Salamat Company) according to the manufacturer's instructions. Inhibition of pyrogallol autoxidation and decrease in the absorbance at 405 nm were used to detect SOD activity. GPx activity was determined by monitoring the level of the consumption of NADPH with decreasing absorbance at 340 nm. The measurement of TAC was conducted by the ferric reducing ability of plasma (FRAP) assay. This assay is based on the reduction of Fe^3+^ to Fe^2+^ by antioxidants present in the sample, with the absorbance monitored at 593 nm (Benzie & Strain, 1996).

### 2.6 Lipid oxidation assay

At day 42, after slaughter, the breast and thigh meat of eight chickens per treatment were removed and used for the lipid oxidation assay. Measuring MDA was performed as a secondary oxidation product based on the thiobarbituric acid method as explained by Botsoglou et al. (1994).

### 2.7 Intestinal microbiota assay

At day 42, eight chickens were slaughtered from each treatment. The small intestine (from the jejunum distal end to the ileocecal junction) was dissected to assess the numeral of total aerobic, coliform, and lactic acid bacteria. Immediately postmortem, the small intestine was longitudinally opened. A sterile knife was used to scrape the digestive contents and mucosal surface, and the ileal contents were then transferred using aseptic technique into sterile tubes. One gram of the homogenized ileal content was diluted from 10^1^ to 10^8^ in sterile phosphate‐buffered saline (PBS). Agar media, plate count agar, MacConkey agar, and MRS agar were used to enumerate total aerobic, coliform, and lactic acid bacteria, respectively. Each duplicate sample dilution was inoculated on agar plates and incubated at 37°C for 24 h before counting. The counts were stated as log_10_ CFU/g.

### 2.8 Histological morphometric assay

At the end of the experimental period, eight chickens per treatment were randomly selected and slaughtered to prepare tissue samples from the small intestine. The small intestine was removed, and 2 cm sections of the duodenum, jejunum (between the entry of the bile duct and Meckel's diverticulum), and ileum were excised, rinsed, and stabilized using physiological serum in 10% saline‐formalin solution for 24 h. Tissue was dehydrated, clarified, and embedded in paraffin in order before cutting into 5‐μm‐thick sections using a rotary microtome. After mounting on glass slides, the sections were stained with both hematoxylin–eosin and alcian blue. The determination of villus height, crypt depth, and the density of goblet cells (per 100 μm villus height) was conducted with an Olympus light microscope using imaging software (Dino Capture 2.0). At last, the ratio of villus length to crypt depth (VH/CD) was calculated.

### 2.9 Immune response assay

At day 9, two chickens per pen were subcutaneously vaccinated (0.2 mL per chicken) with inactivated bivalent NDV and H9N2 avian influenza virus (AIV) vaccines. At day 19, the blood of these chickens was drawn using heparinized syringes to assess the antibody titers for AIV and NDV. A hemagglutination inhibition assay to assess antibody titers was conducted on the collected blood plasma. In addition, at day 28, 0.1 mL of 5% SRBC in sterile PBS was injected into the breast muscle of two chickens per pen. A booster injection was also given per chicken at day 35 to measure the secondary anti‐SRBC antibody response. After 7 days, the blood of each chicken receiving the SRBC was drawn with heparinized syringes, and the plasma was assayed for anti‐SRBC antibody titers via a microhemagglutination test using 96‐well microplates. The assessment methods were carried out according to Sedaghat and Karimi Torshizi (2017).

### 2.10 Lymphatic organs assay

At day 42, eight chickens were slaughtered from each treatment, and the spleen and bursa were removed and quickly weighed after slaughtering. Then, their relative weight percent was determined.

### 2.11 Plasma biochemistry assay

Blood samples were randomly collected from two chickens per pen using heparinized syringes via the jugular vein. Then, the samples were centrifuged within 20 min at 1000 × *g* at room temperature to separate the plasma. The plasma samples were stored at −20°C in Eppendorf tubes until further analysis. Plasma biochemistry measures, such as total protein, uric acid, albumin, globulin, cholesterol, triglycerides, and glucose, were spectrophotometrically analyzed with analytical kits (Pars Azmun Company) and a microplate reader (Stat Fax 3200; Awareness Technology Inc). Ultimately, the ratio of albumin to globulin was determined.

### 2.12 Statistical analysis

This work was performed in a completely randomized design. The obtained data were exposed to ANOVA via the SAS software (SAS 9.4, 2014) GLM Proc. The statistical significance was compared among the means at a 95% significance level through Duncan's multiple‐range test. Results are presented as the mean value.

## 3 RESULTS

### 3.1 Growth performance

There was no difference among trial treatments in ADFI, ADG, and FCR for the different phases with the exception that in the starter phase there was an improvement (*p* = .01) in FCR for the ROE treatment compared to the control group (Table 3).

**TABLE 3:** Effect of the supplemented diet with essential oils on the performance of broiler chickens (42 days of age).

Variables	Treatments					
	CO	PAE1	PAE2	ROE	SEM	*p*‐value
0–10 days						
ADG (g)	14.29	14.32	14.57	14.65	0.171	.86
ADFI (g)	19.63	18.67	19.84	18.28	0.300	.20
FCR(g/g)	1.37^a^	1.30^ab^	1.36^a^	1.25^b^	0.016	.01
11–24 days						
ADG (g)	55.28	61.19	61.95	62.65	1.127	.07
ADFI (g)	78.14	81.06	80.71	80.40	1.095	.80
FCR(g/g)	1.41	1.33	1.30	1.29	0.026	.34
25–42 days						
ADG (g)	78.22	83.92	75.15	73.9	2.421	.49
ADFI (g)	154.38	162.56	158.49	158.58	2.386	.72
FCR(g/g)	2.03	1.94	2.12	2.21	0.069	.58
0–42 days						
ADG (g)	55.35	59.77	56.33	56.04	1.115	.53
ADFI (g)	96.88	101.14	99.55	99.11	1.316	.75
FCR(g/g)	1.77	1.69	1.77	1.78	0.037	.85

*Note*: ^a,b^Means with different letters within the same row differ significantly (*p* < .05).

Abbreviations: ADFI, average daily feed intake; ADG, average daily gain; Co, Control; FCR, feed conversion ratio; PAE1, *Plectranthus amboinicus* essential oil 100 mg/kg; PAE2, *Plectranthus amboinicus* essential oil 200 mg/kg; ROE, rosemary essential oil 100 mg/kg.

### 3.2 Antioxidant activity

In the present study, GPx and SOD activities were elevated in the plasma (*p* < .0001) by both feed additives compared to the control, except 200 mg/kg PAE which showed no significant effect on GPx. In addition, supplemented diets had increased (*p* = .006) TAC. Moreover, all the supplemented diets reduced MDA in both breast (*p* < .0001) and thigh (*p* < .001) when compared to the control group (Table 4).

**TABLE 4:** Effect of the supplemented diet with essential oils on the antioxidant status of broiler chickens (42 days of age).

Variables	Treatments					
	CO	PAE1	PAE2	ROE	MSE	*p*‐value
SOD (U/mL), Plasma	84.03^d^	142.04^a^	113.69^b^	99.36^c^	8.08	<.0001
GP_X_ (U/mL), Plasma	155.62^c^	198.09^a^	157.01^c^	182.31^b^	6.74	<.0001
TAC (mmol/L), Plasma	0.707^b^	0.905^a^	0.884^a^	0.849^a^	0.026	.006
MDA, breast (nmol/L)	37.38^a^	23.03^c^	33.50^b^	22.56^c^	2.45	<.0001
MDA, thigh (nmol/L)	110.06^a^	65.87^c^	73.49^c^	85.44^b^	6.40	.001

*Note*: ^a,b,c,d^Means with different letters within the same raw differ significantly (*p* < .05).

Abbreviations: CO, control; GPx, glutathione peroxidase; MDA, malondialdehyde; PAE1, *Plectranthus amboinicus* essential oil 100 mg/kg; PAE2, *Plectranthus amboinicus* essential oil 200 mg/kg; ROE, rosemary essential oil 100 mg/kg; SOD, superoxide dismutase; TAC, total antioxidant capacity.

### 3.3 Intestinal microbiota

In this study, dietary supplementation with both levels of PAE decreased (*p* < .005) coliform counts. Moreover, both EOs increased (*p* = .029) lactic acid bacteria counts in the ileum (Table 5).

**TABLE 5:** Effect of the supplemented diet with essential oils on intestinal microbiota of broiler chickens (42 days of age).

Variables	Treatments					
	CO	PAE 1	PAE 2	ROE	SEM	*p*‐value
Intestinal microbiota^1^						
Total aerobic bacteria	6.70	6.44	6.25	6.38	0.097	.439
Coliform	4.73^a^	3.46^b^	3.19^b^	4.00^ab^	0.19	.005
Lactic acid bacteria	5.11^b^	5.83^a^	5.90^a^	5.67^a^	0.111	.029

*Note*: ^a,b^Means with different letters within the same row differ significantly (*p* < .05).

Abbreviations: Co, control; PAE1, *Plectranthus amboinicus* essential oil 100 mg/kg; PAE2, *Plectranthus amboinicus* essential oil 200 mg/kg; ROE, rosemary essential oil 100 mg/kg.

^1^ The number of colonies was counted, and all the data are expressed as log_10_ CFU/g.

### 3.4 Intestinal morphology

This study showed that PAE increased (*p* < .036) VH in the duodenum and that all the supplemented EOs increased (*p* < .041) VH in the jejunum. Also, VH (*p* = .023) was increased by 200 mg/kg PAE in the ileum. The ratio of VH to CD was not significant in the different parts of the intestine. Furthermore, the CD was increased (*p* = .022), and the density of goblet cells was decreased (*p* = .046) by ROE and 200 mg/kg PAE in the ileum (Table 6).

**TABLE 6:** Effect of the supplemented diet with essential oils on the performance of broiler chickens (42 days of age).

Variables	Treatments					
	CO	PAE1	PAE2	ROE	SEM	*p*‐value
Duodenum						
Villus height (μm)	1240.54^b^	1373.35^a^	1451.55 ^a^	1338.18 ^ab^	30.940	.036
Crypt depth (μm)	215.77	263.58	289.22	251.96	11.343	.087
VH/CD	5.75	5.24	5.02	5.33	0.141	.382
Goblet cell density (No/100 μm)	10.83	8.50	8.67	9.17	0.348	.058
Jejunum						
Villus height (μm)	1095.81^b^	1216.72^a^	1253.16^a^	1224.34^a^	24.73	.041
Crypt depth (μm)	216.06	231.54	266.64	261.50	8.763	.059
VH/CD	5.11	5.25	4.70	4.68	0.143	.453
Goblet cell density (No/100 μm)	12.67	11.33	9.67	10.33	0.446	.082
Ileum						
Villus height (μm)	885.66^b^	967.22^b^	1081.56^a^	946.94^b^	28.46	.023
Crypt depth (μm)	114.59^c^	158.38^cb^	212.05^a^	163.83^b^	13.84	.022
VH/CD	7.72	6.11	5.10	5.91	0.410	.091
Goblet cell density (No/100 μm)	12.83^a^	11.17^ab^	10.00^b^	10.67^b^	0.383	.046

*Note*: ^a,b^Means with different letters within the same row differ significantly (*p* < .05).

Abbreviations: Co, control; PAE1, *Plectranthus amboinicus* essential oil 100 mg/kg; PAE2, *Plectranthus amboinicus* essential oil 200 mg/kg; ROE, rosemary essential oil 100 mg/kg; VH/CD, villus height divided by crypt depth.

### 3.5 Immune response

In the present study, anti‐NDV antibody titers, with the exception of the 200 mg/kg PAE treatment, increased (*p* < .0001) with feed additives. Total anti‐SRBC antibody titers in supplemented diets were higher (*p* < .0001) when compared to the control group (Table 7). However, IgY and anti‐SRBC antibody titers, anti‐AIV antibody titers, and lymphatic relative weight percent were not significantly affected by the feed additive treatments.

**TABLE 7:** Effect of the supplemented diet with essential oils on the immune response of broiler chickens.

Variables	Treatments					
	CO	PAE 1	PAE 2	ROE	SEM	*p*‐value
21 days						
Anti‐AIV titers (log_2_)	3.02	3.11	3.05	3.08	0.017	.37
Anti‐ND titers (log_2_)	0.33^b^	1.86^a^	0.50^b^	1.39^a^	0.153	<.0001
42 days						
Total anti‐SRBC (log_2_)	2.83^c^	3.24^a^	3.00^b^	2.93^b^	0.034	<.0001
IgY titers (log_2_)	1.79	2.05	1.86	2.07	0.055	.18
Bursa (%)^1^	0.16	0.13	0.16	0.18	0.010	.16
Spleen (%)^1^	0.08	0.09	0.08	0.09	0.004	.86

*Note*: ^a,b,c^Means with different letters within the same row differ significantly (*p* < .05).

Abbreviations: AID, avian influenza disease; Co, control; IGY, immunoglobulin Y; ND, Newcastle disease; PAE1, *Plectranthus amboinicus* essential oil 100 mg/kg; PAE2, *Plectranthus amboinicus* essential oil 200 mg/kg; ROE, rosemary essential oil 100 mg/kg; SRBC, sheep red blood cells.

^1^ As the percentages of live body weight.

### 3.6 Plasma biochemistry

In the current study (Table 8), the total protein values were increased (*p* = .04) in birds fed 200 mg/kg PAE. The globulin also was increased (*p* = .02) by all the feed additive treatments. In addition, cholesterol levels were lowered (*p* = .002) by EOs. The other parameters measured were not statistically different from the control group.

**TABLE 8:** Effect of the supplemented diet with essential oils on plasma biochemistry parameters of broiler chickens (42 days of age).

Variables	Treatments					
	CO	PAE1	PAE2	ROE	SEM	*p*‐value
UA (mg/dL)	5.47	5.68	5.87	5.61	0.09	.46
TP (g/dL)	4.49^b^	4.71^ab^	4.82^a^	4.69^ab^	0.04	.04
Alb (g/dL)	3.81	3.58	3.56	3.58	0.05	.22
Glob (g/dL)	0.68^b^	1.12^a^	1.25^a^	1.10^a^	0.07	.02
A/G^1^	5.60	3.20	2.85	3.25	0.58	.06
Glu (mg/dL)	272.83	258.74	266.79	272.97	2.60	.18
CHL (mg/dL)	158.65^a^	146.24^b^	136.59^b^	145.54^b^	2.21	.002
TG (mg/dL)	151.68	158.34	160.71	150.90	3.18	.64

*Note*: ^a,b^Means with different letters within the same row differ significantly (*p* < .05).

Abbreviations: Alb, albumin; CHL, cholesterol; Co, Control; Glob, globulin; Glu, glucose; PAE1, *Plectranthus amboinicus* essential oil 100 mg/kg; PAE2, *Plectranthus amboinicus* essential oil 200 mg/kg; ROE, rosemary essential oil 100 mg/kg; TG, triglycerides; TP, total protein; UA, uric acid.

^1^ The ratio of Albumin to Globulin.

## 4 DISCUSSION

There have been several factors postulated as being involved in improving health and performance traits by EOs in animals (Brenes & Roura, 2010), including antibacterial and antioxidant effects, and an increase in enzymatic secretion in the gastrointestinal tract. Previous studies have shown positive effects of PA extract on growth performance in white‐leg shrimp (Huang et al., 2022), and similar effects have been shown by thyme oil and ROE (Amouei et al., 2021; Mahgoub et al., 2019) in broiler chickens and quail. However, some researchers (Abbasi et al., 2020; Placha et al., 2019) observed no significant improvements in ADFI, ADG, or FCR when supplementing with thyme oil or ROE. In the current study, there was no noticeable difference among trial treatments, with the exception of an improvement in FCR in the starter phase for the ROE diet.

The level of MDA can be used as an indicator of oxidative stress and antioxidant conditions in the bird, as it is one of the ultimate products of polyunsaturated fatty acid peroxidation in the cells. However, endogenous antioxidants such as SOD, catalase, GPx, and exogenous antioxidants (as phytobiotics) can function synergistically to neutralize oxidative stress and recycle endogenous antioxidants (Singh et al., 2017). As seen in our study, Manjamalai and Grace (2012) and Palani et al. (2010) indicated that PAE and PA extract could increase GPx and SOD activities and decrease MDA value in both plasma and kidneys of mice and rats. Moreover, researchers found that dietary supplementation with ROE increased the activity of SOD and GP_X_ enzymes in the liver of quail (Mahgoub et al., 2019). In contrast, Alagawany and Abd El‐Hack (2015) and Placha et al. (2019) observed no changes in glutathione (GSH), MDA or TAC, and GPx activity when supplementing birds with rosemary powder and thyme oil, respectively.

This study showed that EOs reduced MDA values in the breast and thigh meat. Hashemipour et al. (2013) reported that thymol and carvacrol supplementation made no difference in breast muscle's MDA value, but did in thigh muscle. Also, Placha et al. (2019) expressed that the available thymol in the diet did not affect lipid oxidation and fatty acid composition due to a low residue of it in breast muscle. However, Loetscher et al. (2013) and Abbasi et al. (2020) both revealed that the MDA value of the breast meat was decreased significantly by ROE and thyme oil supplementation when compared to a control group.

The phytobiotic antimicrobial activity is accepted to be due to the lipophilic property of their components permeating the cell membranes and the mitochondria of microorganisms, which inhibits energy metabolism and membrane‐bound electron flow. Thus, a collapse occurs in the proton pump and then a draining of the ATP pool (Adaszyńska‐Skwirzyńska & Szczerbińska, 2017) and finally, cell death. Our results revealed an increase in lactic acid bacteria and a decrease in coliform counts by the assessed feed additives. Masouri et al. (2017) and Mahgoub et al. (2019) observed similar results on lactic acid bacteria and on *E. coli* or coliform counts using *Satureja khuzistanica* essential oil and ROE in chickens and quail, respectively. However, some studies have shown no influence of EOs on lactic acid bacteria (Mohiti‐Asli & Ghanaatparast‐Rashti, 2018) or on both coliform and lactic acid bacteria (Kırkpınar et al., 2011). It has been inferred that EOs are more effective on Gram‐positive than Gram‐negative bacteria (Trombetta et al., 2005); however, in contrast, Aguiar et al. (2015) observed that PAE was more effective on *E. coli* than on *S. aureus*. The chemical composition of EOs is known to be affected by the geographical origin and harvesting period, which may explain the various effects of EOs against Gram‐positive and Gram‐negative bacteria (Brenes & Roura, 2010).

Similar to this study, previous studies have also shown that supplementing with EOs increases villus height and crypt depth (Amer et al., 2021; Du & Guo, 2021) in broiler chickens. Researchers have suggested that EOs may protect the intestine and favor increase in intestinal surface area, via antioxidant activity and improvement of the intestinal bacterial population (Windisch et al., 2008; Zeng et al., 2015). These changes increase the level of absorption and improve intestinal function.

Goblet cells are secretory epithelial cells that secrete mucus. Mucin glycoproteins, as the principal part of mucus, create a preservative barrier to pathogenic bacteria. They also lubricate the intestinal surface and form a layer to prevent damage to cells by endogenous digestive enzymes, gastric acid, and ingested feed (Alemao et al., 2021). Previous experiments showed an increase in the density of goblet cells in chickens with EOs (Liu et al., 2018) or no effect with mixed EOs and organic acids (Abdelli et al., 2020). However, as in our work, Amer et al. (2021) reported a decrease in the density of goblet cells in the small intestine with a treatment containing oregano essential oil and a medium‐chain fatty acid. Du and Guo (2021) observed a significant reduction in the expression of the Mucin2 gene in chickens challenged with *C. perfringens*, as well as a numerical reduction in the density of small intestinal goblet cells when feeding an essential oil containing thymol and carvacrol. They stated that this phenomenon could be from a direct effect of the essential oil or via an indirect effect through the change in the intestinal microbial population.

Silitonga et al. (2015) indicated that PA extract increased IgM and lysozyme activity in the serum of rats. This agrees with the current study which showed that anti‐NDV antibody titers were influenced by adding thyme oil and ROE to the diet (Abo Ghanima et al., 2020; Nahed et al., 2020; Saleh et al., 2014). In contrast, Attia et al. (2017) and Rostami et al. (2018) obtained no effect of dietary supplements with thyme oil and rosemary powder on NDV. Moreover, Hashemipour et al. (2013) observed that total anti‐SRBC antibody titers could be elevated by supplementing carvacrol and thymol in the diet of broiler chickens. However, Videla et al. (2020) found no difference in antibody titers against SRBC when supplementing the diet with thymol. Similar to these studies, Saleh et al. (2014) and Nahed et al. (2020) showed no significant differences in the relative weight percent of lymphatic organs (spleen and bursa) or in anti‐AIV antibody titers.

Our study results were in line with the other researchers that showed an increase in total protein and globulin values (Attia et al., 2017; Gumus et al., 2017; Mahgoub et al., 2019). Herve et al. (2018) stated that EOs might affect serum total protein and globulin due to their antioxidant and immunostimulatory activity that can improve the immune response. Furthermore, in the present study, the cholesterol levels were lowered by EOs in the broiler chickens. Gumus et al. (2017) and Abo Ghanima et al. (2020) reported a similar result with thyme oil and ROE added to the diets of quail and laying hens. Meeran et al. (2015) observed that thymol reduced 3‐hydroxy‐3‐methyl‐glutaryl‐coenzyme A reductase in the liver of rats. The latter study showed an increase in serum HDL cholesterol, which resulted in increased lecithin cholesterol acyl transferase, a cholesterol‐esterifying enzyme, in the liver. These results describe the effect of this essential oil constituent increasing the transfer of cholesterol from the circulating system to the liver.

Previous studies have inferred a relationship between antioxidant activity, intestinal microbial population, and increased intestinal efficiency due to increased intestinal surface area. Improvement of the expressed traits can improve growth performance. Nevertheless, in our research, growth performance was not affected. It should be noted that EOs and their compounds can show their effects in conditions of environmental tension, stress, and disease in a more prominent way than in nonchallenging conditions.

## 5 CONCLUSION

In this study, we tried to investigate PAE as a newer supplement compared to the more commonly used ROE. We observed that PAE and ROE improved antioxidant activity, intestinal microbiota, intestinal morphology, immune response, and plasma biochemistry parameters. PA is widely used as a medicinal and culinary herb. Therefore, it may be used in the poultry industry, in the same way as thyme and oregano, to improve the growth and health of the animal. In the future, more studies need to be carried out to elucidate the mechanisms of action of this herb in poultry.

## AUTHOR CONTRIBUTIONS

**Saied Hosseinzadeh:** Conceptualization (equal); data curation (equal); formal analysis (equal); investigation (equal); methodology (equal); validation (equal); visualization (equal); writing – original draft (equal); writing – review and editing (equal). **Farid Shariatmadari:** Conceptualization (equal); funding acquisition (equal); methodology (equal); project administration (equal); resources (equal); supervision (equal); validation (equal); writing – review and editing (equal). **Mohammad Amir Karimi Torshizi:** Conceptualization (equal); formal analysis (equal); methodology (equal); software (equal); validation (equal); visualization (equal); writing – review and editing (equal). **Hamed Ahmadi:** Conceptualization (equal); data curation (equal); methodology (equal); project administration (equal); validation (equal); visualization (equal). **Dawn V Scholey:** Writing – review and editing (equal).

## CONFLICT OF INTEREST STATEMENT

The authors declare that they do not have any conflict of interest.

## ETHICS STATEMENT

The study protocol was evaluated and accepted by the Tarbiat Modares University's Ethics Committee for animal use.

## Data Availability

The data that support the findings of this study are available on request from the corresponding authors (above). The data are not publicly available due to privacy or ethical restrictions.
